# Psychosocial impact of COVID-19 2 years after outbreak on mental health of medical workers in Iran

**DOI:** 10.1186/s43045-022-00276-z

**Published:** 2023-01-16

**Authors:** Pirhossein Kolivand, Saereh Hosseindoost, Zahra Kolivand, Zeinab Gharaylou

**Affiliations:** 1 Departmant of health economics, school of medicine, Shahed University, Tehran, Iran; 2 Pain Research Center, Neuroscience Institute, Tehran University of Medical Sciences, Tehran, Iran; 3 Brain and Spinal Cord Injury Research Center, Neuroscience Institute, Tehran University of Medical Sciences, Tehran, Iran; 4 Tehran University of Medical Sciences, Tehran, Iran; 5 Shefa Neuroscience Research Center, Khatamolanbia Hospital, Tehran, Iran

**Keywords:** Depression, Anxiety, Stress, COVID-19

## Abstract

**Background:**

The COVID-19 pandemic had a substantial influence on the mental health of healthcare workers. This study investigated general health status, the prevalence, and the severity of depressive spectrum and anxiety-related disorders. It evaluated the association between various factors and depression, anxiety, and stress among healthcare workers in the Khatam-Alanbia Hospital in Iran, after 2 years since the corona virus disease 2019 (COVID-19) pandemic.

**Results:**

In this online cross-sectional study, 409 participants were selected and given a questionnaire about demographic, personal, and clinical characteristics as well as stressors related to COVID-19. The participants completed the General Health Questionnaire (GHQ-28) and the 42-item Depression, Anxiety, and Stress Scale (DASS-42) to report depression, anxiety, and stress/tension levels. We found that the overall incidence of depression, anxiety and stress among health care workers during the COVID-19 pandemic was 44.25%, 50.62%, and 43.76%, respectively. Participants with severe to very severe depression, anxiety and stress accounted for 19.2%, 26.6%, and 18.2% of the sample, respectively. Being female was associated with higher odds of depression, anxiety, and stress.

**Conclusions:**

Two years after the COVID-19 outbreak, health workers are still showing a significant level of depression, anxiety, stress, and remarkable signs of psychological distress. The situation of a health care worker is worrying. The long-term psychological implications of infectious diseases should not be ignored. Mental health services could play an essential role in rehabilitation.

## Background

The severe acute respiratory syndrome coronavirus (SARS-COVID) that induces severe acute respiratory syndrome causes the coronavirus disease 2019 first appeared as clusters of strange respiratory tract infections in Wuhan, China, in December 2020 [1]. Since later, the disease has spread throughout China and to other parts of the world. The World Health Organization defined on March 11, 2020, COVID-19 as a global pandemic [2]. Beginning in February 2020, COVID-19 spread among tourists to the local populace in Iran.

The COVID-19 virus tends to spread in “waves of infections,” which is explained by the frequency curve in Iran. However, after 2 years of restrictions, people became accustomed to their new surroundings. As we learn more about the virus, the hope for a period of stability in daily life is increased by the vaccine program. Despite this, the pandemic’s ongoing presence continues to influence people’s mental health. Numerous reports on the public’s mental health during the COVID-19 outbreak have been released over the previous 2 years [3–6].

However, after the COVID-19 outbreak, there is a need to pay closer attention to human mental health. A thorough assessment study of this subject is still crucial. Psychological distress in the populace has been observed during infection epidemics due to the disease’s recurrent peaks; this manifests as a variety of symptoms, including depression, insomnia, stress, worry, rage, impatience, and emotional exhaustion. Medical professionals have observed higher prevalence rates of depression, anxiety, sleeplessness, obsessive–compulsive and somatization symptoms, and posttraumatic stress symptoms [7, 8]. Stress among healthcare workers during the COVID-19 pandemic is correlated with higher anxiety [9]. The risk of exposure to COVID-19-positive individuals, gender, organic disorders, and other characteristics have made them more likely to experience sadness, anxiety, and insomnia [10, 11]. Healthcare professionals are more susceptible to depression, anxiety, stress, and posttraumatic stress symptoms whether they have a history of physical symptoms similar to those of the COVID-19 infection [12, 13]. Despite being aware of its limitations, this study aims to evaluate the respondents’ current state of mental health 2 years after COVID-19 was first identified worldwide. This study examined how demographic factors and prior exposure to COVID-19 affected the differences in mental disorders associated with COVID-19. In this study, we examine the incidence and severity of depression, anxiety, and stress in healthcare professionals and assess the relationships among different variables (demographic, personal, and clinical characteristics; stressors associated with COVID-19; and general health status, depression, anxiety, and stress among healthcare professionals following the picks of COVID-19 in Iran).

## Methods

The aim of the study was to assess the prevalence and severity of stress, anxiety, and depression in the healthcare profession as well as the relationships between various variables. Healthcare was chosen as the subject of this research because it is important for researchers all over to know how the COVID-19 outbreak has affected this group psychologically. This online survey was carried out between September 23, 2021, and October 22, 2021, starting about three weeks after the fifth selection of COVID-19 finished in Iran.

This study was conducted in Khatam-Alanbia Hospital)Tehran Province, Tehran, Vali-Asr St., Rashid Yasemi St.). Khatam-Alanbia Hospital was used for quarantine in COVID pandemic. Total number of health care workers was 2000 persons. The number of beds and clinics were 700 and 42 respectively. The total number of hospital staff was 2000 and, 20.45% (409 persons) of these health workers participated in this study. The healthcare personnel contact with COVID-19 was defined to the following online guidance: https://www.cdc.gov/coronavirus/2019-ncov/hcp/guidance-risk-assessment-hcp.html. The study sample was conducted online because isolation has replaced closeness as the new standard in relation to the COVID-19 outbreak. With permission from the hospital management, the staff sent an email inviting individuals to take part in the study. The online survey form’s settings were modified to prevent repeated submissions from the same participant by enabling the “limited answers with one per person” option in Google Forms. healthcare professionals might participate in the study if they fulfilled the following criteria: (1) minimum age of 18; (2) no previous history of psychotic disorders, or alcohol dependence. The email that served as their invitation provided information on the study's protocols to participants. Participants’ completion and submission of the online questionnaire responses were considered their informed consent.

We obtained information on two clinical aspects: a history of physical and psychological health. We assessed a participant to have a pre-existing medical condition if they self-reported having hypertension, diabetes, chronic lung disease, heart disease, endocrine disorders, nervous system disorders, renal diseases, or cancer. The self-reported psychiatrist characterized the pre-existing psychiatric condition as including depression and anxiety disorders. We show the classifications of the groups and descriptions of each variable in Table 1.

**Table 1:** Demographic and personal characteristics, clinical factors, and COVID-19-related stressors among the participants

Variables	Categories	Number	Percent
Age	< 35 years old	202	49.4
	36–54 years old	190	46.4
	> 54 years old	17	4.2
Gender	Male	177	43.2
	Female	232	56.7
Marital status	Married	273	66.7
	Unmarried	136	33.2
Education level	Primary education	72	17.6
	Secondary education	109	26.7
	Post-secondary education	228	55.7
Employment status	Full-time employed	374	91.44
	Part time employed	35	8.56
Contact with patients infected with COVID-19^a^	No	303	74.08
	Yes	106	25.92
Existing comorbidities connected with increased risk of severe illness caused by COVID-19?	No	353	86.3
	Yes	56	13.69
Pre-existing psychiatric illnesses?	No	400	97.7
	Yes	9	2.2
History of COVID-19 in relatives?	No	115	28.12
	Yes	294	71.88
History of COVID-19 in person?	No	219	53.55
	Yes	190	46.45

Primary education (without academic education), secondary education (bachelor’s and master’s academic education), post-secondary education (doctorate and specialization education)

^a^Coronavirus disease -2019

A self-reported questionnaire was used to gather information on demographic and personal traits, clinical factors, and stresses connected to COVID-19. The questionnaire was developed using data from earlier studies on the psychological effects of infection outbreaks like SARS on the general population [14–16]. The self-reported questionnaire included the demographic and personal characteristics (such as age, gender, marital status, and level of education), clinical factors (such as a history of prior medical or psychiatric illness), and having a history of COVID-19 in person or in a relative that have been linked to psychological complications in previous studies. Despite not receiving complete validation, the questionnaire was created by two psychiatrists and a public health expert. The General Health Questionnaire (GHQ-28) and the 42-item Depression, Anxiety, and Stress Scale (DASS-42) were also given to the participants to assess depression, anxiety, and stress. Google Forms was used to conduct this online, cross-sectional investigation. A web-based survey administration tool called Google Forms enables the online posting and completion of questionnaires by selected respondents. After that, an automated spreadsheet entry process can be used to add the acquired data.

For public mental health screening, the GHQ-28 is a commonly used self-administered questionnaire [17]. The four subscales are depression, social dysfunction, sleep problems, and somatic complaints. With a maximum potential score of 84 and a minimum score of 0, the Likert-type scale was used to rate each item from 0 to 3. A psychiatric disorder was identified by a total score of under 23 and a subscale score of under 7. Analysis shows that the GHQ-28 has strong reliability and validity in developing nations. The Persian version of the GHQ-28 has been shown to have good internal reliability (Cronbach’s alpha = 0.70–0.90) [18, 19].

The DASS-42 was used to evaluate the participant's stress, anxiety, and depression symptoms [20]. This self-reported test has 42 items divided into three categories: stress, anxiety, and depression. The fourteen items that make up each subscale are graded on a Likert scale from 0 to 3 (where 0 means “did not apply to me at all,” 1 means “applicable to me to some degree or occasionally,” 2 means “applied to me largely, or a majority of the time,” and 3 means “applied to me very much, or most of the time”). The sum of the item scores is used to get the overall score for each subscale; a higher score indicates severe symptoms. For a case study, the thresholds for depression, anxiety, and stress are 9, 7, and 14, respectively. The ratings for depression severity also fall into the following ranges: (1) mild depression ranges from 10 to 13, (2) moderate depression from 14 to 20, (3) severe depression from 21 to 27; and (4) extremely severe depression from 28 to 42. The following are the possible scores for the level of anxiety: (1) moderate anxiety = 10–14, (2) severe anxiety = 15–19, (3) extremely severe anxiety = 20–42, and (4) mild anxiety = 8–9. The scores for different stress levels are as follows: mild stress is scored between 15 and 18, moderate stress is between 19 and 25, severe stress is between 26 and 33, and highly severe stress is between 34 and 42. With Cronbach's alpha values for the depression, anxiety, and stress subscales of 0.71, 0.79, and 0.81, respectively, it has been shown that the Iranian version of the DASS-42 has internal reliability. Furthermore, concept validity is high [21].

The IBM SPSS Statistics program, version 25, was used to conduct data analyses (SPSS Inc., Chicago, IL, USA). Depression, anxiety, and stress incidence and severity were estimated, along with other descriptive statistics for demographic and personal characteristics, clinical variables, and stressors associated with COVID-19. No one’s values were missing. reported as percentages, and frequencies were categorical variables. Then, simple logistic regression analyses were used to calculate the crude odds ratios (ORs), where the absence of depression, anxiety, or stress was coded as 0 (reference), and the presence of depression, anxiety, or stress was coded as 1. This calculation allowed for the individual association between various demographic and personal characteristics, clinical factors, and stressors related to COVID-19 and depression, anxiety, and stress (dependent variables). Following that, factors with *p* < 0.1 were added to several logistic regression models to determine their adjusted ORs for forecasting stress, anxiety, and depression (dependent variables). The presence of depression or anxiety was coded as 1, and the absence of both was coded as 0. The Hosmer–Lemeshow test, where *p* < 0.05 indicated model fit, was used to evaluate the multiple logistic regression model’s fit. Stepwise logistic regression analyses (both forward and backward) were performed to confirm the significant predictors of depression, anxiety, and stress. All *p* values were calculated using a two-sided significance level of *p* 0.05.

## Results

### Participant characteristics

The online survey was finished by 409 people altogether. Table 1 provides a summary of the participants’ demographic and personal traits, clinical considerations, staff involved with COVID-19 in the workplace, personal experience with COVID-19, and relations. Nearly three-quarters of the participants (*n* = 232, 56.7%) were female, with a median age of 30–45 years (*n* = 266, 66.7%). Most participants (*n* = 337, 82.3%) had a postgraduate education, and about two-thirds were married (*n* = 273, 66.7%).

According to the analysis of the clinical factors, more than one-seventh of the participants (*n* = 56, 13.6%) had a pre-existing medical condition, although only a tiny percentage (*n* = 9, 2.2%) had a pre-existing psychiatric condition.

According to the DASS-42 scores, 44.25% of participants had depression, 11.7% had mild depression, 13.2% had moderate depression, and 19.2% had severe depression. Additionally, 50.62% of the individuals reported having anxiety, of whom 10.2% had mild symptoms, 13.6% had moderate symptoms, and 26.6% had severe to extremely severe symptoms. 43.76% of participants reported feeling stressed; of these, 11.2% reported mild stress, 15.1% reported moderate stress, and 18.2% reported severe to highly severe stress (Table 2).

**Table 2:** Psychological characteristics of the participants

Variables	Categories	Number	Percent
Depression	No	228	55.74
	Yes	181	44.25
Severity of depression	None	228	55.74
	Mild	48	11.7
	Moderate	54	13.2
	Severe	53	12.9
	Extremely severe	26	6.3
Anxiety	No	202	49.38
	Yes	207	50.62
Severity of anxiety	None	202	49.38
	Mild	42	10.2
	Moderate	56	13.6
	severe	72	17.6
	Extremely severe	37	9
Stress	No	230	56.23
	Yes	179	43.76
Severity of stress	None	230	56.23
	Mild	46	11.2
	Moderate	62	15.1
	Severe	49	11.9
	Extremely severe	22	5.37

### Analysis of GHQ-28

The mean score in the GHQ-28's overall analysis was 21.1 (SD 14.2). Respondents (*n* = 161 or 39.3%) reached the threshold for mild mental illnesses (24 points). The factors that affected the GHQ-28 outcome are illustrated in Table 3. Women scored significantly higher than males in both the overall interpretation and the GHQ-28 subscales (*p* = 0.006). Additionally, there was a statistically significant correlation between the mean GHQ-28 scores and marital status, pre-existing comorbidities linked to an elevated risk of severe illness brought on by COVID-19, and history of COVID-19 in the respondents' families (*p* ≤ 0.05). The mean GHQ-28 scores did not statistically correlate with the level of education, workplace, employment status, or personal history of COVID-19 (*p* ≤ 0.05).

**Table 3:** A detailed analysis of the effect of individual factors on GHQ-28 score and its subscales

Variables	Categories	GHQ-28^a^	Somatic symptoms	Anxiety/sleep disorder	Social dysfunctions	Depression	χ^2^ (*P*)
	Total	21.1 (14.2)	6.2 (3.62)	6.08 (4.9)	3.72(3.7)	5.02 (3.67)	
**Gender**	Female	23.8 (13.9)	7.1 (3.6)	7.09 (5.06)	3.9 (3.7)	5.5 (3.5)	95.62 (0.006)
	Male	17.5 (13.9)	5.1 (3.4)	4.7 (4.5)	3.4 (3.7)	4.2 (3.7)	
Marital status	Unmarried	23.3 (15.5)	6.72 (4.1)	6.7 (5.1)	4.30 (4.14)	5.55 (3.9)	81.12 (0.04)
	Married	20.04 (13.4)	6.08 (3.5)	5.7 (4.8)	3.45 (3.4)	4.7 (3.5)	

^a^General Health Questionnaire

### The associations among clinical factors and participant characteristics

Multivariate logistic regression analysis indicated that the probability of anxiety (ORa = 1.07, 95% CI 1.03–1.12), stress (ORa = 3.140, 95% CI 1.964 to 5.022)) and depression (ORa = 1.787, 95% CI 1.129 to 2.829) risk increased with being female (Table 4). The multiple logistic regression model for anxiety reported a Nagelkerke *R*^2^ of 0.129 (*p* < 0.001), and the Hosmer–Lemeshow goodness-of-fit test (*χ*2 = 2.687, df = 8, *p* = 0.952), for stress a Nagelkerke *R*^2^ of 0.150 (*p* < 0.001), and the Hosmer–Lemeshow goodness-of-fit test (*χ*2 = 8.292, df = 8, *p* = 0.405), and for depression a Nagelkerke *R*^2^ of 0.155 (*p* < 0.001), and the Hosmer–Lemeshow goodness-of-fit test (*χ*2 = 5.631, df = 8, *p* = 0.689) indicated good model fit.

**Table 4:** Association between gender and anxiety, depression, and stress among the participants

Variables	Categories	Crude OR^a^ (95% CI)	β^b^	_SE_^b^_ß_	_*p* value_^b^	Adjusted OR^b^ (95% CI)
Depression	Male	1				1.941(1.259) to 2.992)
	Female	1.787 (1.129 to 2.829)	0.663	0.221	0.003	
Stress	Male	1				3.430 (2.191 to 5.368)
	Female	3.140 (1.964 to 5.022)	1.232	0.229	0.000	
Anxiety	Male	1				2.672 (1.721 to 4.149)
	Female	2.795 (1.752 to 4.459)	0.983	0.224	0.000	

## Discussion

This study considered the burden of general mental health and the prevalence and severity of general health disorders, depression, anxiety, and stress among healthcare workers after the 2 years of the outbreak of COVID-19. Then, the relationship between distinct demographic, personal, and clinical characteristics and COVID-19-induced depression, anxiety, and stress was determined. The results of this study indicated that 2 years after the outbreak of COVID-19 in Iran, in 39.3% of the health care workers participants of Khatam-Alanbia Hospital, general mental health is somewhat impaired. The prevalence rates of depression, anxiety, and stress among the healthcare workers were 44.25%, 50.62%, and 43.76% respectively. Also, after the fifth peak of the COVID-19 outbreak, the range of depression, anxiety and stress remained between 12.2 and 50.4%, 13.0 and 44.6%, and 29.1 and 71.5%, respectively. When we compared the severity of the psychological symptoms of our study with the results of other studies, the prevalence of severe to very severe depression (19.2%), severe to very severe anxiety (26.6%), and severe to very severe stress (18.2%) in our study was similar to them. The results of two cohort studies in the Asia–Pacific region that used DASS-21 as a screening tool for the psychological symptoms associated with the COVID-19 epidemic are similar to our report [22, 23]. A nationwide cross-sectional experiment between Dutch intensive care nurses revealed that the first COVID-19 pick had a high influence on the mental health of intensive care nurses, enhancing the risk for dropout and imperiling the continuity of care. In this study, the prevalence rates of symptoms of depression, post-traumatic stress disorder, anxiety, and need for recovery were documented by 18.6%, 22.2%, 27.0%, and 41.7%.of the participants, respectively. Working in the hospital, being scared of contaminating relatives and experiencing inadequate numbers of coworkers associated with higher mental symptoms, while having been on vacation was associated with lowered depression signs and need for recovery [24]. Another study investigated the mental health of Critical Care Registered Nurses supplying direct patient care during the early peak of the COVID-19 pandemic in Canada. In the experiment, the participants reported mild to severe depression (57%), stress (54%), anxiety (67%), as well as significant symptoms of post-traumatic stress disorder (38%). Indeed, critical care nurses revealed psychological disorders associated with supplying care to COVID-19 patients during the initial surges of the pandemic [25]. Yarong Ma and colleagues examined the severity of stress and possible correlates between the health care professionals searching online mental health care during the COVID-19 outbreak. The sample overall indicated moderate levels of stress, which 24% suffering from anxiety and 38% recognized as depressed. Moreover, the staffers at intensive care units or in departments of respiratory medicine exhibited remarkably more elevated stress than workers in other units [26].

During the first wave of the SARS-COVID-2 pandemic, the research in Poland showed a mean GHQ-28 score of 31.74 ± 16.93 [14]. The evidence collected from India presents a compromise of mental disorders with a mean GHQ-28 score of 24.18 ± 14.00 in 42.16% of respondents [27]. In our study, the criterion for mental disorders (≥ 24 points) was met by 39.3% of respondents. Regardless of the different populations and the study method, the incidence rates in our study appear lower than the previous similar studies. However, in comparison with a survey conducted on Iranian nurses in 2017, the increase in mental health burden (39.3 versus 30.2%) was observed in our study. Comparing the results of our survey with the results of other studies that reported before the epidemic (despite different methods and study groups), it can be concluded that the COVID-19 pandemic maybe had a significant impact on the mental state of the respondents even after 2 years. Evidence for changes in the mental health of health care workers during the wave of the COVID-19 pandemic in Argentina, regarding the starting point in anxiety levels, showed that there was increasing anxiety outcome among healthcare workers as the pandemic progresses [28]. Iran is a country whose people have experienced many stressful events in recent years, such as floods, earthquakes, and economic sanctions. The higher rate of mental health problems in Iran compared to other countries and its doubling in recent years maybe because of the cumulative adverse effects of stressful events on mental health [29].

Our findings indicated that the female gender is significantly more disposed to depression, stress, and anxiety among the healthcare workers. The main factors influencing the decline of mental health during COVID-19 include female sex, a low level of education, and the coexistence of chronic diseases [30]. However, the longitudinal study showed that increasing age, living with the elderly, and concerns about workload and risk of infection were associated with higher odds of depression and anxiety among physicians over 1 year after the COVID-19 outbreak [31]. As in our study, gender, marital status, pre-existing comorbidities, and history of COVID-19 in the respondents’ families were significantly associated with the higher GHQ-28 mean scores, but did not affect the severity of depression and anxiety and stress. In some study reports, women are more likely to tend towards mental disorders in response to stressful situations. However, the significant prevalence of women (81.8%) among responders may influence the result of the study’s analysis [14, 32]. In most online surveys, most participants are female, because women are more enthusiastic to participate in the surveys. In our study, however, the proportion of men and women was almost the same. A study reported that 1 year after the SARS outbreak, being a woman and a healthcare worker were risk factors for poor psychological change. Females showed higher levels of stress, depression, and anxiety and they had more severe posttraumatic stress symptoms [33]. In another study, according to GHQ-12 scores, females showed three times more psychological complications than male SARS survivors [34]. This result is compatible with our findings that females had more depression, stress, and anxiety symptoms 2 years after the COVID-19 outbreak.

Working in a hospital during the COVID-19 epidemic may result in psychological trauma for the health care workers [35]. However, 80 to 90% of individuals exposed to stress do not develop posttraumatic stress disorder (PTSD) [36]. Nevertheless, several aspects may increase the specific expected risk correlated with COVID-19, including concerns about dealing with a disease of unknown cause before identifying the COVID-19 corona virus and the disease’s rapid international spread and substantial mortality. Studies of healthcare workers in China during the peak of the COVID-19 outbreak indicated that the front-line medical staff and those working in medical units were being highly exposed to COVID-19 patients and feared infection, which predisposed them to depression [37]. Those who worked in the epicenter of the COVID-19 outbreak had a higher likelihood of developing depression. In contrast, according to our study, after 2 years of corona outbreak and passing the fifth peak in Iran, workplace and exposure to COVID-19 patients are independent of the psychological impact of the COVID-19, but other agents such as gender, marital status, and underlying diseases are essential factors. Applying the results that healthcare workers are not predisposed to mental health disorders can be consistent with the conclusion that having more years of experience in health care work was accompanied by a lower incidence of psychiatric disorders.

This research did not include health care workers who quit work or did not work during the study period due to long-term disability. As a result, mental health problems that were severe enough to result in persistent disability were not considered. Studies of excessive disability after the COVID-19 outbreak be helpful but are not available. Therefore, although the results are consistent with the interpretation that 2 years from the COVID-19 outbreak did not increase the risk for psychiatric disorders in health care workers because of workplace stressors, caution is required. The finding of this study highlights the importance of health care workers’ attention to providing task training in dealing and preparing for a pandemic and other emergent disease in a health care environment. Limitations of the present research include the fact that self-report obtained our data. We could not compare individual participants because the questionnaires were anonymous to ensure confidentiality. However, since the population is captive and the previous study in Iran was not different on core demographic variables, it is reasonable to conclude that stress levels of COVID-19 remained persistent but did not increase over the 2 years.

## Limitations

Some limitations of this experiment must be addressed. This study only evaluated the general health status in the Khatam-Alanbia Hospital in Iran. The general health status of other health centers and other countries is not included. Moreover, there are no baseline or control groups for comparison prevalence of symptoms with them. There is no reliable report on the prevalence rates of our results in health workers of Khatam-Alanbia Hospital before the COVID-19 pandemic.

## Conclusions

The current survey provides insights into the probable long-term adverse psychological effects of infectious diseases. Our study shows that anxiety, depression, and stress levels remained significant 2 years after the outbreak instead of abating with time. It could be suggested that psychological supplies could be necessary for the rehabilitation phase and should not be forgotten as we face the developing new episode of the delta-COVID-19 virus.

The health worker may be classified as a vulnerable population because they are disclosed to COVID-19 in their work environment and exposed to developing psychological situations. It is essential that the aspects of their experiences be deeper than aspects that cannot be addressed with a quantitative method. Efforts are needed to optimize working situations, such as empowering health staffers to rejuvenate physically and mentally and reduce workload. The prevention strategies should concentrate on decreasing the stress, for example, creating psychological support efficiently available and organizing regular support sessions.

## Data Availability

The data of this research are available from the corresponding author upon request.

## Abbreviations

DASS-42: Depression Anxiety and Stress Scale-42
GHQ-28: General Health Questionnaire
SARS-COVID: Severe acute respiratory syndrome coronavirus
